# The wild strawberry kinome: identification, classification and transcript profiling of protein kinases during development and in response to gray mold infection

**DOI:** 10.1186/s12864-020-07053-4

**Published:** 2020-09-14

**Authors:** Hui Liu, Wei Qu, Kaikai Zhu, Zong-Ming ( Max) Cheng

**Affiliations:** 1 College of Horticulture, Nanjing Agricultural University, Nanjing, 210095 China; 2 College of Forestry, Nanjing Forestry University, Nanjing, 210037 China; 3 Department of Plant Sciences, University of Tennessee, Knoxville, 37996 USA

**Keywords:** Strawberry protein kinases, Gene duplication, Receptor-like kinases (RLKs), Transcript profiling, *Botrytis cinerea* infection

## Abstract

**Background:**

Protein kinases (*PKs*) play an important role in signaling cascades and are one of the largest and most conserved protein super families in plants. Despite their importance, the woodland strawberry (*Fragaria vesca*) kinome and expression patterns of *PK* genes remain to be characterized.

**Results:**

Here, we report on the identification and classification of 954 *Fragaria vesca PK* genes, which were classified into nine groups and 124 gene families. These genes were distributed unevenly among the seven chromosomes, and the number of introns per gene varied from 0 to 47. Almost half of the putative *PKs* were predicted to localize to the nucleus and 24.6% were predicted to localize to the cell membrane. The expansion of the woodland strawberry *PK* gene family occurred via different duplication mechanisms and tandem duplicates occurred relatively late as compared to other duplication types. Moreover, we found that tandem and transposed duplicated *PK* gene pairs had undergone stronger diversifying selection and evolved relatively faster than WGD genes. The GO enrichment and transcriptome analysis implicates the involvement of strawberry *PK* genes in multiple biological processes and molecular functions in differential tissues, especially in pollens. Finally, 109 *PK*s, mostly the receptor-like kinases (RLKs), were found transcriptionally responsive to *Botrytis cinerea* infection.

**Conclusions:**

The findings of this research expand the understanding of the evolutionary dynamics of *PK* genes in plant species and provide a potential link between cell signaling pathways and pathogen attack.

## Background

Protein kinases (*PK*) are a large and widely distributed protein superfamily found in prokaryotes and eukaryotes and comprise one of the largest and most conserved protein gene super-families in plants. They play important roles in various signaling pathways via phosphorylation of serine, threonine, and tyrosine amino acids in target proteins. The first plant protein kinases to be identified and characterized were from *Pisum sativum* in 1973 [1]. In *Arabidopsis thaliana*, there are more than 1000 *PKs*, collectively called a kinome [2], and many *PK*s in other plant species have been reported, including soybean [3], tobacco [4], cotton [5] and rice [6]. In general, *PK* gene families are bigger in plant genomes than in those of animals [7, 8]. For example, in humans, *PK*s only account for 1.7% of the coding sequence [9] whereas in *Arabidopsis* and rice, they account for ~ 4 and 5%, respectively [6, 10]. The number of *PK* genes can vary widely between plant species. In the pineapple genome, the kinome contains 758 *PK* members, whereas in soybean there are over 2000, twice the number of Arabidopsis [3, 10]. Notably, these kinomes are a rich resource for conducting comparative analyses to predict putative functions and to understand the evolutionary dynamics of *PK* genes in plant species.

Protein kinases all share a common catalytic domain, comprised of about 230–280 amino acids [11]. Based on the conservation and phylogenetic analysis of this catalytic domain, plant kinome is divided into five major groups [11]. Using this criterion, Hanks and Hunter [9] classified the entire PK superfamily into nine groups. Subsequently, Lehti-shiu and Shiu defined the PKs from 25 plant species in nine groups and 115 families, including group PKA-PKG-PKC (AGC), calcium- and calmodulin-regulated kinase (CAMK), casein kinase 1 (CK1), cyclin-dependent kinases (CMGC), mitogen-activated protein kinases (MAPK), glycogen synthase kinases and cyclin-dependent like kinases, sterility (STE), tyrosine kinase-like kinases (TKL), receptor-like kinase (RLK), plant-specific and finally other, a group of kinases that could not be classified easily into the previous groups [12].

Woodland strawberry (*Fragaria vesca; Rosacea*) is one of the most widely distributed indigenous species in the northern hemisphere [13]. As one of the progenitors of the cultivated octoploid strawberry, *Fragaria* × *ananassa* [14], it serves as a model for this economically important species. The genome of the woodland strawberry is ~ 240 Mb in size with seven pairs of chromosomes (2*n* = 2*x* = 14) [15]. With both genomic and transcriptomic data available, comprehensive transcriptomic and proteomic studies are possible. Some woodland strawberry PK genes have been characterized and shown to be involved in abiotic and biotic stress responses including MAPKs [16], AMP-activated protein kinase (AMPK) [17], leucine-rich repeat receptor-like protein kinase (LRR-RLK) [18], and calcium-dependent protein kinase (CDPK) [19].

Here, we report on the identification and in silico characterization of 954 putative woodland strawberry *PK* genes, which were categorized into nine groups and 124 gene families based on the kinase domain. We determined the structure and chromosomal distribution of the *PK* genes, as well as made predictions on the subcellular localization of the putative PK proteins. We investigated the evolutionary dynamics of this gene family in woodland strawberry, including selection pressure on different types of duplicated gene pairs. Finally, we conducted an in silico analysis on *PK* gene expression patterns in different tissues across development and in response to *Botrytis cinerea* attack. Thus, we present a comprehensive analysis of the *PK* genes found in the woodland strawberry genome and their developmental expression patterns and responses to biotic stress.

## Results

### Genome-wide identification and classification of protein kinases in woodland strawberry

Using an HMM approach, a total of 954 putative woodland strawberry *PK* genes were identified (Additional file 1: Table S1 and Additional file 2: Figure S1), all of which fell into one of nine groups, AGC, CAMK, CK1, CMGC, Plant-specific, RLK, STE, TKL, and “Others”. Out of all the groups, the RLK group had the most members, which accounted for 67.0% of the total *PK* genes. All *PK* members were further classified into 124 families (Additional file 4: Table S3), out of which, 39 families contained only one member. The RLK-Pelle_DLSV family was the largest, with 128 members.

### The properties of woodland strawberry kinome

To characterize the 954 strawberry *PK*s, the gene structure, kinase domain and predicted subcellular localizations of their putative protein translations were determined (Additional file 5: Table S4). Strikingly, 920 strawberry *PK* genes (96.4%) had two or more kinase domains. Whereas, the remainder PK genes only had one kinase domain, and these genes were distributed in 18 different families (Additional file 6: Table S5).

In the analysis of *PK*s gene structure, it was found that the number of introns per gene varied widely from 0 to 47, with an average intron number of six. *mrna23790* (RLK-Pelle_DLSV) was the *PK* with the most introns. Out of the 954 *PK* genes, 144 (15.1%) lacked introns. In others, 197 (20.6%) of the *PK*s contained more than ten introns, while 34 (3.6%) others contained more than 20 introns. At kinase family level, members in CMGC_SRPK, RLK-Pelle_LRR-VII-1, RLK-Pelle_LRR-VII-2, RLK-Pelle_LRR-VII-3, and RLK-Pelle_RLCK-X families had the same number of introns. However, the exon/intron boundary in some *PK* genes in some families was highly variable. Among 34 members in the STE_STE11 family, 11 were intronless, whereas each of the remaining 23 family members contained four to 30 introns. Based on the phylogenetic relationships of these genes in the STE_STE11 family, all of the members could be clearly divided into two clusters based on the number of introns-clusters without introns and clusters that are intron-rich (> 3 introns per gene; Additional file 2: Figure S1). These data suggest that the kinase families had their own evolutionary expansions subsequent to divergence from one another.

**Fig. 1:**
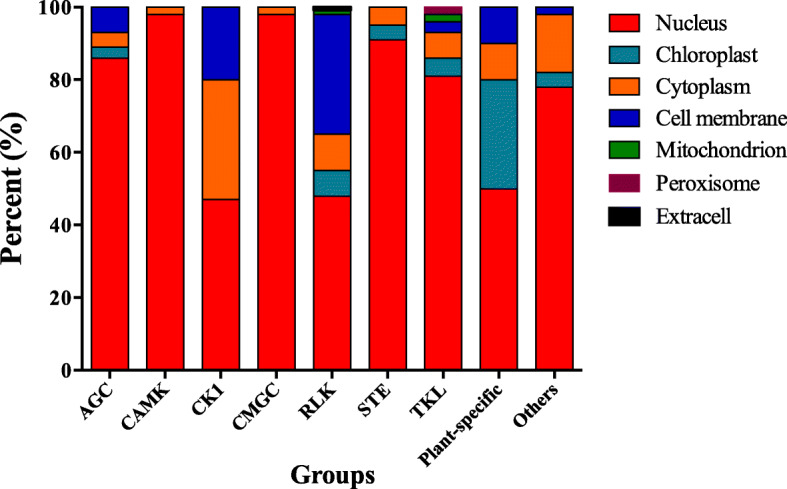
The predicted subcellular localization of woodland strawberry protein kinases in each kinase group. Different colors represent different cellular compartments

To gain further insights into the potential functions of the woodland strawberry PK proteins, the subcellular localization of each amino acid translation was predicted using Plant-mPLoc. The result indicated that 58.4% of the PKs were predicted to localize to the nucleus and 24.6% were predicted to localize to the cell membrane (Fig. 1). The remaining kinase genes were predicted to localize to the chloroplast, cytoplasm, mitochondrion, peroxisome, and extracell, respectively (Additional file 3: Table S2). The PKs in different kinase groups were predicted to localize to different cellular compartments. About 100% (59/59) CAMK and 97.0% (64/66) CMGC members were predicted to localize to the nucleus, whereas 45.4% (290/639) RLK members were predicted to localize to the cell membrane. Among all the kinase families, 23 kinase families were predicted to have the same subcellular locations for all members.

### Different duplication types among woodland strawberry PKs

Gene duplication plays a crucial role in the evolution of plant genomes and diversification of protein function [20], and can occur via whole-genome duplication (WGD) and single-gene duplication events [21]. Single-gene duplication can be further divided into tandem duplication (TD), proximal duplication (PD), transposed duplication (TRD), and dispersed duplication (DSD) [20]. The woodland strawberry kinome had 78 WGD events with 145 *PK* genes, that involved 90 RLK kinase genes (Additional file 7: Table S6), and 141 strawberry PK genes underwent 80 TD events, among which, 72 events occurred in the RLK group. We identified 58 PD events with 105 PK genes, a total of 193 TRD events with 318 PK genes from 71 gene families, and 839 DSD genes with 918 PK genes from 119 gene families. Additional file 7: Table S6 shows different duplication patterns drove the expansion of woodland strawberry *PK* genes.

**Fig. 2:**
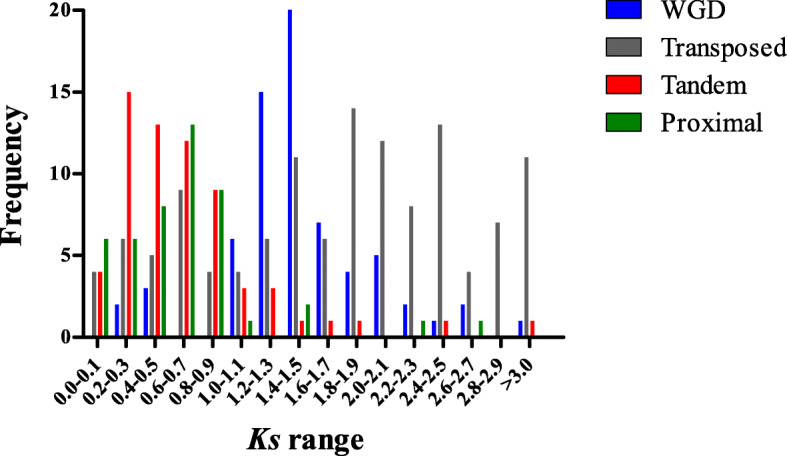
The distribution of *Ks* ratios frequency among different duplication events in strawberry kinome. The X-axis denoted average *Ks*, and Y-axis denoted frequency

In order to estimate the time of different duplication types in the *PK* genes, synonymous substitution (*Ks*) rates of the duplicated gene pairs were determined. The *Ks* frequency of WGD kinase genes peaked at 1.4 to 1.5, much greater than the peak range of 0.2 to 0.3 in TD genes (Fig. 2). Among the TRD events, the *Ks* frequency peaked at 1.8–1.9, which was the greatest peak value in all the duplication types. The TRD of *PK* genes occurred before the WGD-resulted kinase genes. However, the tandem duplication *PK* genes appeared relatively later than the other types of kinase duplications.

**Fig. 3:**
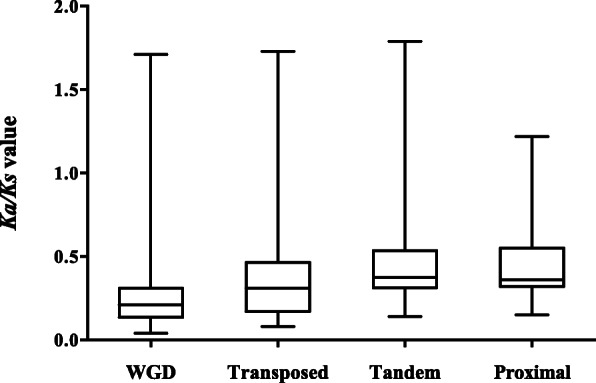
The *Ka/Ks* values of different duplication events in strawberry kinome. The bars at the top and bottom of the whiskers mean maximum and minimum values; the top and bottom of the box represent third and first quartiles; bar in the box mean median values

To estimate selective pressure on strawberry PKs between different duplication types, *Ka/Ks* values were calculated for each gene pair. A *Ka/Ks* ratio less than 1 indicates purifying selection, a *Ka/Ks* ratio equal to 1 implies neutral selection, while *Ka/Ks* value greater than 1 indicates positive selection [22]. Almost all gene pairs, including all the types of duplicates, had a *Ka/Ks* value of less than 1 (Fig. 3 and Additional file 8: Table S7). The WGD genes had significant lower *Ka/Ks* values in median, average, and quartile than TD and TRD genes (*t*-test, *P* < 0.01). These results suggest that WGD-derived gene pairs have narrower distribution of *Ka/Ks* values, WGD genes evolve slower and are under weaker selection pressure than the gene pairs derived from other duplication types.

### Chromosomal distribution of woodland strawberry PKs

**Fig. 4:**
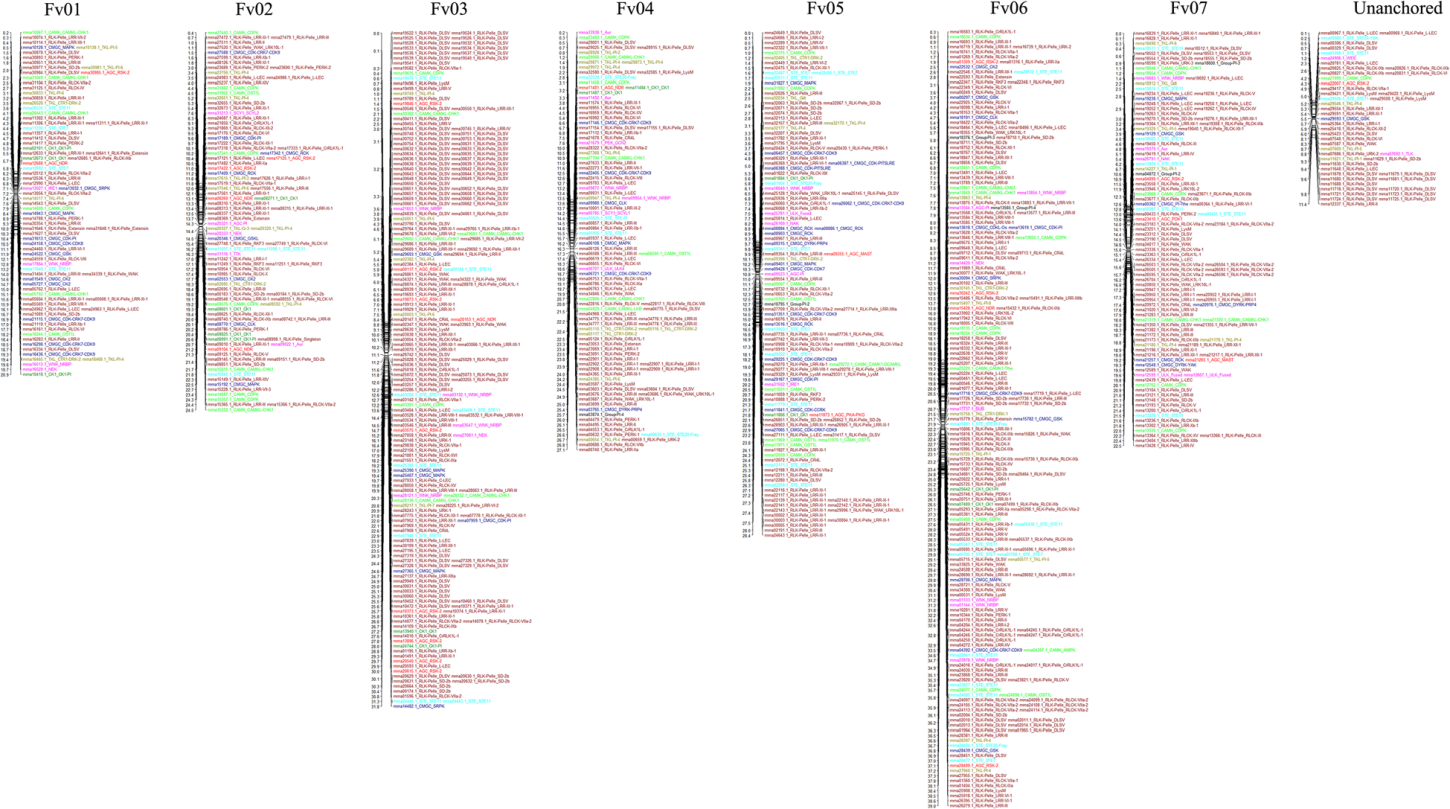
Chromosomal locations of all the kinase genes in woodland strawberry. Gene IDs with corresponding family names were indicated to the right of each chromosome, and related information on gene location is listed on the left

To determine the chromosomal distribution of woodland strawberry *PK*s, a total of 907 genes were mapped, and it was found that they are unevenly distributed across the seven chromosomes. Chromosome 6 and 3, which is the longest, harbored the two largest numbers of kinase genes, 197 and 191 genes, respectively. Chromosome 1 contained the fewest with 81 *PK* genes (Fig. 4). The strawberry *PK* members in the same group were generally clustered together on different chromosomes. For example, the largest numbers of CAMK and STE members were distributed on chromosome 6, whereas the greatest number of RLK members was located on chromosome 3 (Additional file 3: Table S2). Although the gene number of strawberry PKs was partly related to chromosome length, the uneven distribution of PKs in different groups was also found between different chromosomes.

### Functional prediction of woodland strawberry PK genes

**Fig. 5:**
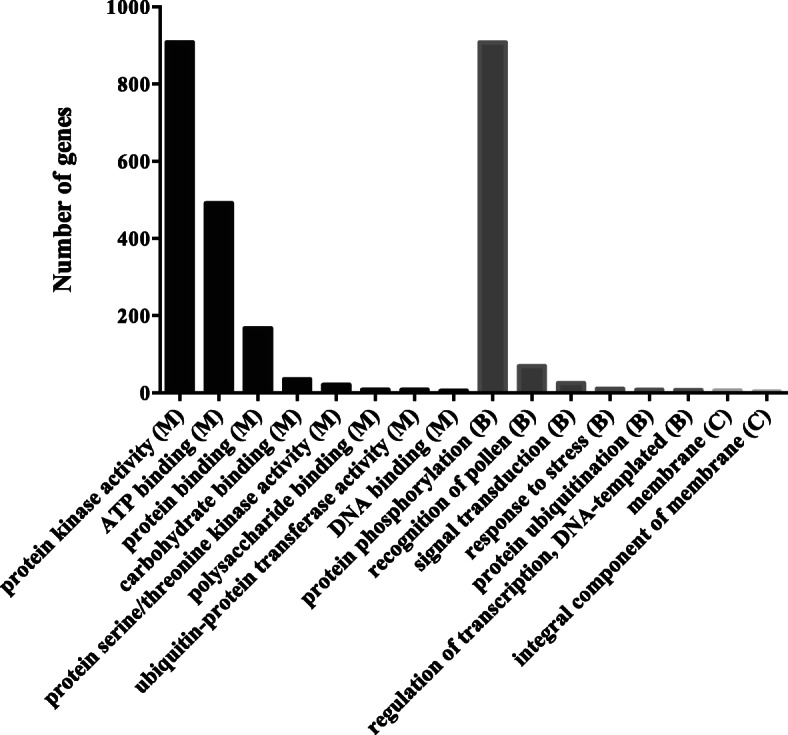
Gene Ontology (GO) analysis of strawberry PKs

**Fig. 6:**
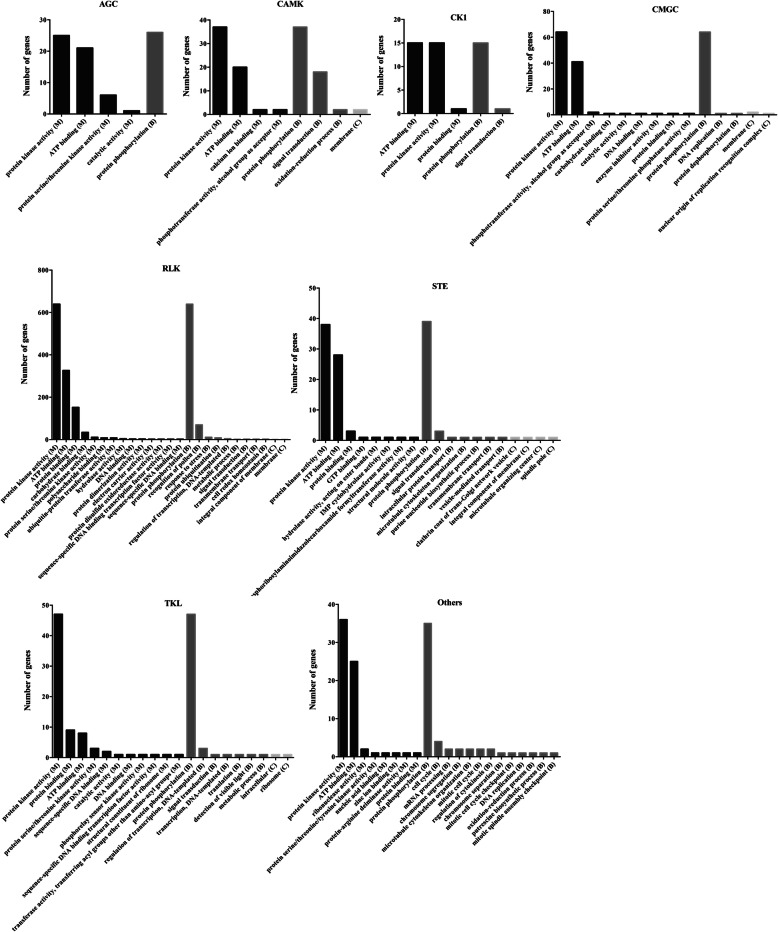
The strawberry genes in each kinase group enriched in (**a**) biological process (**b**) molecular process (**c**) cellular component

To determine the putative functions of woodland strawberry *PK*s, the GO annotations for all the genes were examined and were assigned and classified into three main GO categories: biological process, molecular function, and cellular component (Fig. 5). Functional GO terms for the PK genes were also analyzed. The tops three GO terms in molecular function were assessed as “protein kinase activity”, “ATP binding”, and “protein binding”. The woodland strawberry *PKs* were enriched in GO terms of epigenetic processes, such as “protein phosphorylation, in GO terms of development, “recognition of pollen”, and in GO terms of signaling cascades, “signal transduction”. All the *PKs* were enriched in cellular component of membrane. Furthermore, the strawberry *PKs* in each kinase group enriched in biological process and molecular function was found similar (Fig. 6). However, the *PKs* in the RLK kinase group were enriched in terms of “response to stress”.

### Expression patterns of woodland strawberry PKs in different tissues during development

**Fig. 7:**
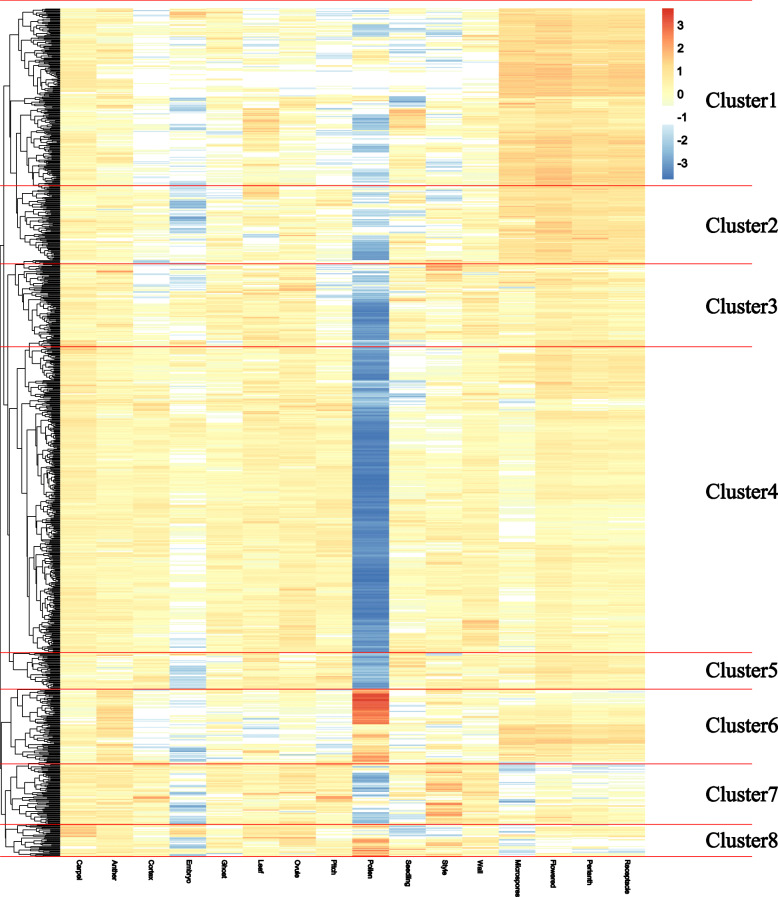
A heatmap illustrating the expression data of 952 strawberry PKs in 16 different strawberry tissues and developmental stages. The color scale represents expression levels, with red indicating high expression levels and blue indicating low levels

**Fig. 8:**
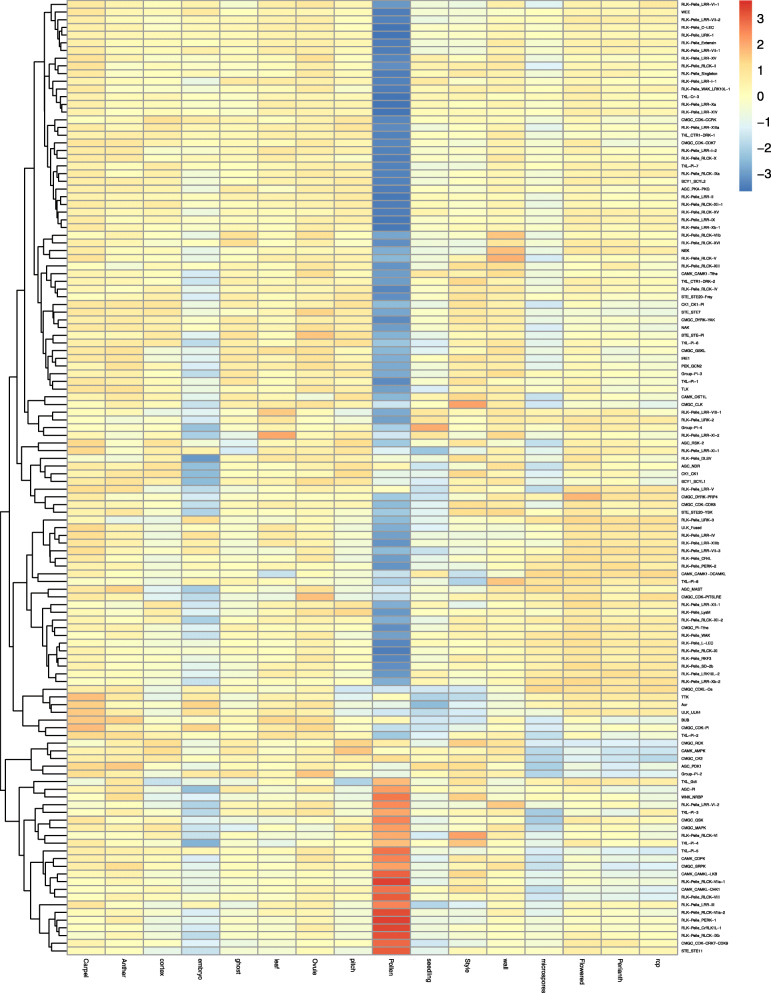
A heatmap demonstrating the expression data of 124 different strawberry kinase families in 16 different strawberry tissues and developmental stages. The color scale represents expression levels, with red indicating high expression levels and blue indicating low levels

In order to explore the expression patterns of strawberry *PK* genes in different tissues, an in silico analysis of the transcriptomic data from carpel, anther, cortex, embryo, ghost, leaf, ovule, pith, pollen, seedling, style, wall, microspores, flowers, perianth, and receptacle was conducted [23]. Based on the heatmap cluster analysis of PK expression, the 952 woodland strawberry *PK* genes were classified into eight clusters (Fig. 7 and Additional file 9, 10, 11, 12, 13, 14, 15 and 16: Figure S2-9). Cluster 1 contained 204 *PK*s, with numerous genes exhibiting high expression in microspores, flower, perianth, and receptacle, and low expression in pollen (Additional file 9: Figure S2). In cluster 2, most *PK* genes also had high levels of expression in microspores, flower, perianth, receptacle, but with low levels of expression in embryo and pollen (Additional file 10: Figure S3). The *PK* genes in cluster 3, 4, and 5 showed significant down-regulation in pollen (Additional file 11, 12 and 13: Figure S4-S6). However, in cluster 6, most genes had high levels of expression in pollen (Additional file 14: Figure S7). The GO analysis of the *PKs* in each cluster supported the results. The woodland strawberry *PKs* in cluster 1–6 were all enriched in GO terms of “recognition of pollen” (Additional file 17: Figure S10). Interestingly, the *PK* genes that had high expression levels in microspores, flower, perianth, and receptacle had low expression levels in pollen. To further explore the relationship between woodland strawberry *PK* gene families and expression patterns in pollen, a heatmap was constructed (Fig. 8). Where most *PK* families had low expression in pollen, RLK − Pelle_RLCK−VIIa− 1, RLK − Pelle_RLCK−VIIa− 2, and RLK − Pelle_PERK− 1 kinase families were significantly up-regulated in pollen. Taken together, these results suggest that *PK* families have distinct expression patterns with regards to tissue type.

### RNA-seq analyses of woodland strawberry *PK* genes in response to gray mold infection

**Fig. 9:**
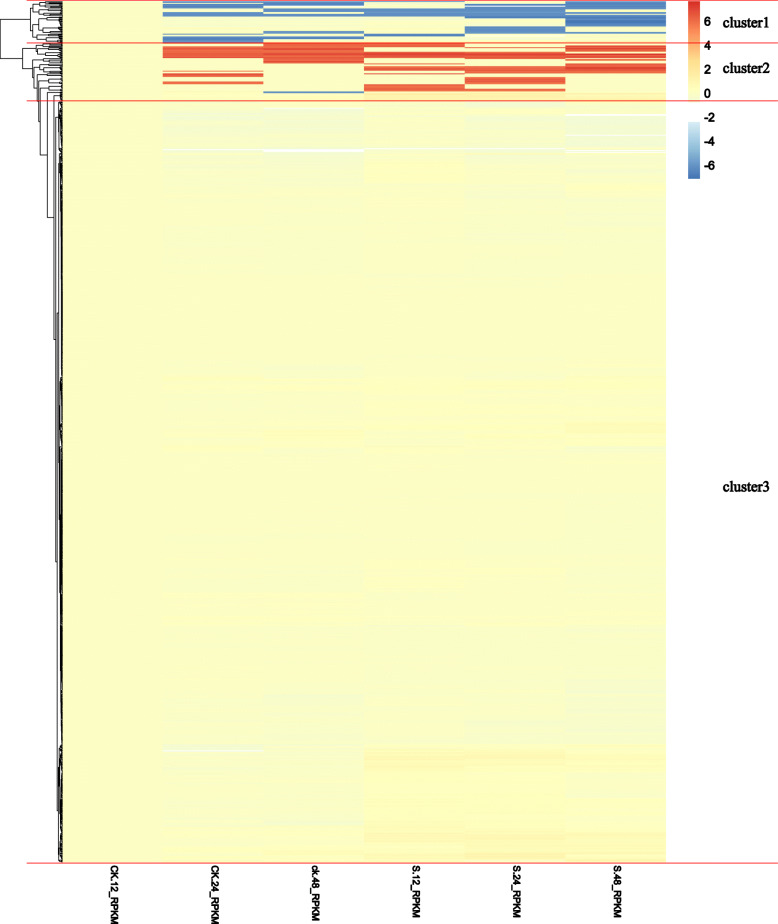
A heatmap of the expression data of all the strawberry kinase genes in response to gray mold. The color scale represents expression levels, with red indicating high expression levels and blue indicating low levels

*Botrytis cinerea* is the causal agent of gray mold disease, which causes serious economic loss in fresh strawberry. In order to investigate whether the strawberry PK genes are associated with the defense of mature strawberry fruits against this pathogen, we mined the transcriptome data of mature fruits infected with *B. cinerea.* There were 109 kinase genes (in cluster 1 and 2) that exhibited differential expression patterns. These genes showed significant up- or down-regulation in response to *B. cinerea* attack (Fig. 9)*.* Interestingly, among the 46 down-regulated genes (cluster 1), 38 (82.6%) were from the RLK kinase group (Additional file 18: Figure S11). Moreover, there were 50 RLK genes (79.4%) among the 63 up-regulated kinase genes (cluster 2) (Additional file 19: Figure S12). However, most woodland strawberry *PK* genes in cluster 3 showed little changes and variations comparing with the control upon *B. cinerea* infection (Additional file 20: Figure S13). The heatmap indicated that the 109 strawberry kinase genes in cluster 1 and 2 played important roles in response to *B. cinerea*. In addition, the genes in the RLK kinase group associated with strawberry gray mold disease responses.

## Discussion

### The RLK group is the largest group of PKs in woodland strawberry kinome

Protein kinases transfer a phosphoryl group from ATP to specific amino acids in target proteins, which acts as a switch to activate or inactive target proteins, thus affecting the downstream cascades of biological processes [24]. The RLK kinase group, the largest group of protein kinases, has a variety of extracellular domains that excert function in a large number of processes, from cell wall interactions to disease resistance to developmental control [25]. Over 600 RLK genes are found in Arabidopsis, making up > 2% of its genome, and almost 61% of the Arabidopsis kinome [26]. The proportion of RLKs is also over 50% of the kinome in other species including pineapple (63.3%), soybean (67.4%), and grapevine (74.6%) [3, 27, 28]. In this study, a total of 639 RLK genes were identified, accounting for about 67% of the woodland strawberry kinome, which is consistent with the species mentioned above. The strawberry RLK group contained 58 kinase families, approximately 46.8% in all strawberry kinase families. Among these kinase families, 15 (25.9%) of the strawberry RLK families contained more than ten members. Moreover, the RLK-Pelle_DLSV and RLK-Pelle_LRR-XI-1 families were the largest, which contained 128 and 60 members, respectively. Because only two and three RLK members are found in *Chlamydomonas reinhardtii* and *Volvox carteri*, respectively, the expansion of RLK group has likely occurred after the divergence of land plants [25].

### Different duplication patterns drive the expansion of woodland strawberry kinome

Gene duplication is a primary source of genetic novelty, morphological diversity, and speciation, which is forcing the evolution of plant species [29]. Gene duplication events are divided into five different types: WGD, TD, PD, TRD, and DSD [30]. Previous studies have shown that the expansion and functional diversification of protein kinase genes have been facilitated by gene duplication. Arabidopsis has experienced at least two recent WGDs [31]. The protein kinases have different degrees of functional diversification due to different gene duplication through segmental and tandem duplications [10]*.* Segmental duplication events were the main cause for the expansion of the soybean kinome [3]. Segmental, tandem, or whole-genome duplication events have been key in the expansion of the gene families in both the grapevine and pineapple kinomes, especially in the RLK group [27, 28].

In this study, 937 strawberry *PK* genes experienced duplication events. Almost all *PK* genes in the woodland strawberry have arisen or contributed to gene duplication. A total of 145 strawberry *PK* genes (15.2%), including 90 *RLK* kinase genes (14.1%), were duplicated and retained during WGD (Additional file 7: Table S6). It appears that 141 strawberry *PK* genes (14.8%) have undergone tandem repeat duplication, including 126 RLK genes (19.7%). A total of 318 PK genes (33.3%) were identified, among which 194 RLK genes (30.4%) arose from transposed duplication. The transposed duplication can promote significant changes in gene structure faster than other gene duplication types [32]. Environmental pressure can promote the divergence of duplicated genes, to adapt to dramatic environmental changes because of the frequent occurrence of transposed duplication [30]. Transposed duplicates are consistent with both their antiquity and the nature of their evolution, with novel copies potentially being separated from *cis*-regulatory sequences at the original site and/or exposed to different ones at the new site. The WGD (15.2% PKs) and TD (14.8% PKs) events also played critical roles in the expansion of the strawberry *PK*s. For the *RLK* group, the transposed and tandem repeats provided more opportunities for members of this group to diverge. In contrast to WGD, tandem duplications have taken place much more frequently and are responsible for more of the gene copy number and allelic variation within a population [33]. In a previous study, it was suggested that tandem duplications tend to associate with stress response genes [34].

The *PK* distribution among these duplication events in different duplication types indicated that tandem duplications occurred more recently than other duplication events. Most of the strawberry *PK* tandem duplications had a *Ka/Ks* < 1, which was greater than other duplication types. The “younger” duplicates in tandem duplication type were subjected to stronger diversifying selection and had a faster evolutionary rate.

### The strawberry kinase genes responded to gray mold disease infection

Given their involvement in signaling cascades, protein kinases are heavily implicated in a wide variety of biological processes, including biotic and abiotic stress response in plants [24, 25]. Most of the recent expansion of the *Arabidopsis RLK* genes were reported to be associated with defense/resistance responses [26]. In the woodland strawberry kinome, 109 PK genes were differentially expressed (DEGs) at 24 and 48 h after inoculation as compared to 12 h with *B. cinerea*. In this study, 88 of the *PK* genes belonged to the RLK group (Additional file 18-19: Figure S11-12), suggesting that members of this group play a major role in the woodland strawberry response to this pathogen. This is consistent with the fact that 290 (33.7%) woodland strawberry RLK members were predicted to be localized in the cell membrane. RLKs have a variety of extracellular domains that function as the initial sensors for pathogen molecular signatures and subsequently activate cell wall interactions to initiate disease responses [25, 35]. Previous studies reported that pathogen recognition were linked to transcriptional reprogramming by CDPK/CPK and MAPK cascades [36–38], and the genes reported here will be of interest to elucidate and characterize the underlying biochemistry and molecular biology of the disease response in woodland strawberry.

## Conclusion

A total of 954 putative strawberry protein kinase genes were identified and classified into nine groups and 124 gene families. These genes were distributed unevenly among the seven chromosomes. Almost half of the PKs were predicted to localize to the nucleus and membrane. Transposed duplication played a greater role than other duplication types in the expansion of strawberry *PK*s. Tandem duplication of *PK* genes emerged relatively late in the evolutionary history compared with other types of duplications, and were subjected to stronger positive selection, suggesting a faster evolutionary rate than WGD and TRD-derived genes. The strawberry *PK* gene families demonstrated differential tissue expression patterns, especially in regards to pollen. Additionally, 109 *PK*s showed significant up- or down-regulation in response to *B. cinerea*, 88 of which were *RLK* genes. This research provides insights into the evolution and putative function of woodland strawberry *PK*s, and will provide a foundation for future studies concerning the woodland strawberry kinome, and its associated members, in the functional mechanisms underlying the plant’s response to biotic and abiotic stressors.

## Methods

### Identification and classification of woodland strawberry protein kinases

The predicted proteome for the woodland strawberry was downloaded from Phytozome v12.1 [39]. The proteome was subsequently subjected to a comprehensive search for putative PKs using HMMER v3.1 with an e-value cutoff < 1.0 using the Hidden Markov models (HMMs) Pkinase (Pkinase (PF00069) and Pkinase_Tyr (PF07714)) that were downloaded from Pfam [40]. To improve the accuracy of the putative predictions, the presence of a kinase domain in each of the candidate *PK* genes was verified using Pfam and SMART [41]. A Perl script was used to extract the sequence for each *PK* and to remove duplicates to produce a final list of non-redundant woodland strawberry *PK* genes and a comprehensive kinome.

### Sequence alignment and phylogenetic analysis of strawberry protein kinases

Full-length amino acid translations of the woodland strawberry *PK* genes were aligned using MUSCLE in MEGA X using default settings [42]. A phylogenetic tree was generated using the evolutionary model maximum likelihood (ML) with FastTree v2.1.10 [43, 44].

### Chromosomal locations and intron numbers

The chromosomal positions of the predicted *PK* genes were retrieved from the woodland strawberry database [39], and their locations were mapped to the corresponding chromosomes using MapChart v2.3 software [45]. Gene structures were extracted from the general feature format (GFF3) file using TBtools v0.58 [46].

### Subcellular localization prediction

To provide useful insights into functions of proteins in various cellular organelles, we predicted protein subcellular localization for the woodland strawberry putative *PK* translations using Plant-mPLoc (http://www.csbio.sjtu.edu.cn/bioinf/plant-multi/) [47]. The predictor was powerful and flexible. The input sequence should be in the FASTA format.

### Identification of gene duplication events in woodland strawberry kinome

The duplication events for woodland strawberry *PK*s were retrieved from the Plant Duplicate Gene Database (PlantDGD, http://pdgd.njau.edu.cn:8080) [30]. Tandem duplicates were defined as at least two genes separated by five or fewer genes, and located on the same chromosome within a 100-kb region. Proximal duplication events were defined as gene pairs that were on the same chromosome but separated by less than ten genes. The transposed duplication pairs had to meet the condition of one member of the pair had to exist at the ancestral locus and the other at a non-ancestral locus [21].

### GO analysis of PK genes in woodland strawberry

To report the predicted functions of woodland strawberry *PK* protein translations, the gene ontology (GO) annotations for strawberry PKs were downloaded from the Gene Ontology Consortium (http://www.geneontology.org/) [27].

### Calculation of *Ka*, *Ks* and *Ka/Ks* values

To estimate selection pressure on woodland strawberry *PK* gene pairs, the nucleic acid sequences were aligned using ClustalX 2.0 [48]. Perl scripts were then used to calculate the rate of non-synonymous (*Ka*) and synonymous substitutions (*Ks*), along with the ratio of *Ka* to *Ks* (*Ka/Ks*) for each gene pair [28].

### RNA-Seq expression analysis

Genome-wide transcriptome data from 42 different tissues and development stages of woodland strawberry, “Hawaii 4”, were downloaded from Strawberry Genomic Resources [23, 49–51] and an in silico analysis was conducted to determine differential *PK* gene expression in different tissues across the developmental stages of woodland strawberry. The data were filtered using Trim_galore, a high throughput sequence quality control analysis tool [52]. Then, the filtered reads were mapped to the reference genome by using HISAT2 [53]. The reads of each gene were counted by Subread-featureCounts with default parameters [54]. The differentially expressed genes (DEGs) among the samples were then identified by using the edgeR package [55]. The false discovery rate (FDR) ≤0.01 and an absolute value of the | logFC | ≥2 were used as thresholds to evaluate the significance of gene expression differences. Heatmaps were generated using the heatmap package in R (v3.4.3) [56]. Additionally, to explore the relationship between strawberry *PK* gene expression in response to the pathogen *B. cinereal* with respect to time, we conducted a similar analysis of the expression data from mature strawberry fruits infected with *B. cinerea* at 12, 24, and 48 h post-infection [57]. The 12 h time point was used as the comparative control and heatmaps were generated as described as above.

## Supplementary information

**Additional file 1: Table S1.** Kinase domain annotation of typical woodland strawberry protein kinases.

**Additional file 2: Figure S1.** The phylogenetic tree of all the strawberry protein kinase genes and their intron-exon structures.

**Additional file 3: Table S2.** Sub-family classification of woodland protein kinases and their related information.

**Additional file 4: Table S3.** Gene numbers of 124 kinase families.

**Additional file 5: Table S4.** List of all strawberry protein kinases containing multiple domains.

**Additional file 6: Table S5.** List of strawberry protein kinases containing multiple kinase domains.

**Additional file 7: Table S6.** List of all the duplicated strawberry protein kinases.

**Additional file 8: Table S7.** *Ka/Ks* values of strawberry duplication gene pairs.

**Additional file 9: Figure S2.** A heatmap of the expression data of strawberry kinase genes in cluster 1 in 16 different strawberry tissues and developmental stages.

**Additional file 10: Figure S3.** A heatmap of the expression data of strawberry kinase genes in cluster 2 in 16 different strawberry tissues and developmental stages.

**Additional file 11: Figure S4.** A heatmap of the expression data of strawberry kinase genes in cluster 3 in 16 different strawberry tissues and developmental stages.

**Additional file 12: Figure S5.** A heatmap of the expression data of strawberry kinase genes in cluster 4 in 16 different strawberry tissues and developmental stages.

**Additional file 13: Figure S6.** A heatmap of the expression data of strawberry kinase genes in cluster 5 in 16 different strawberry tissues and developmental stages.

**Additional file 14: Figure S7.** A heatmap of the expression data of strawberry kinase genes in cluster 6 in 16 different strawberry tissues and developmental stages.

**Additional file 15: Figure S8.** A heatmap of the expression data of strawberry kinase genes in cluster 7 in 16 different strawberry tissues and developmental stages.

**Additional file 16: Figure S9.** A heatmap of the expression data of strawberry kinase genes in cluster 8 in 16 different strawberry tissues and developmental stages.

**Additional file 17: Figure S10.** The strawberry protein kinase genes in each cluster enriched in (A) biological process (B) molecular process (C) cellular component.

**Additional file 18: Figure S11.** A heatmap of the expression data of the strawberry kinase genes in cluster 1 response to gray mold.

**Additional file 19: Figure S12.** A heatmap of the expression data of the strawberry kinase genes in cluster 2 response to gray mold.

**Additional file 20: Figure S13.** A heatmap of the expression data of the strawberry kinase genes in cluster 3 response to gray mold.

## Data Availability

All the genomes were obtained from Phytozome (https://phytozome.jgi.doe.gov/). Genome-wide transcriptome data were downloaded from Strawberry Genomic Resources (http://bioinformatics.towson.edu/strawberry/).

## Abbreviations

PK: Protein kinase
AGC: PKA-PKG-PKC
CAMK: Calcium- and calmodulin-regulated kinase
CK1: Casein kinase 1
CMGC: Cyclin-dependent kinase (CDK), mitogen-activated protein kinase (MAPK), glycogen synthase kinase (GSK) and CDC-like kinase (CLK)
MAPK: Mitogen-activated protein kinase
STE: Sterility
TKL: Tyrosine kinase-like kinase
RLK: Receptor-like kinase
AMPK: AMP-activated protein kinase
LRR-RLK: Leucine-rich repeat receptor-like protein kinase
CDPK: Calcium-dependent protein kinase
HMMs: Hidden Markov models
ML: Maximum likelihood
GFF: General feature format
GO: Gene ontology
*Ka*: Non-synonymous substitutions
*Ks*: Synonymous substitutions
WGD: Whole-genome duplication
TD: Tandem duplication
PD: Proximal duplication
TRD: Transposed duplication
DSD: Dispersed duplication
DEG: Differentially expressed gene
