# Supplementary figures and images for: The wild strawberry kinome: identification, classification and transcript profiling of protein kinases during development and in response to gray mold infection
Source: BMC Genomics. 2020 Sep 14;21:635. doi: 10.1186/s12864-020-07053-4 (PMC7490889; doi:10.1186/s12864-020-07053-4)

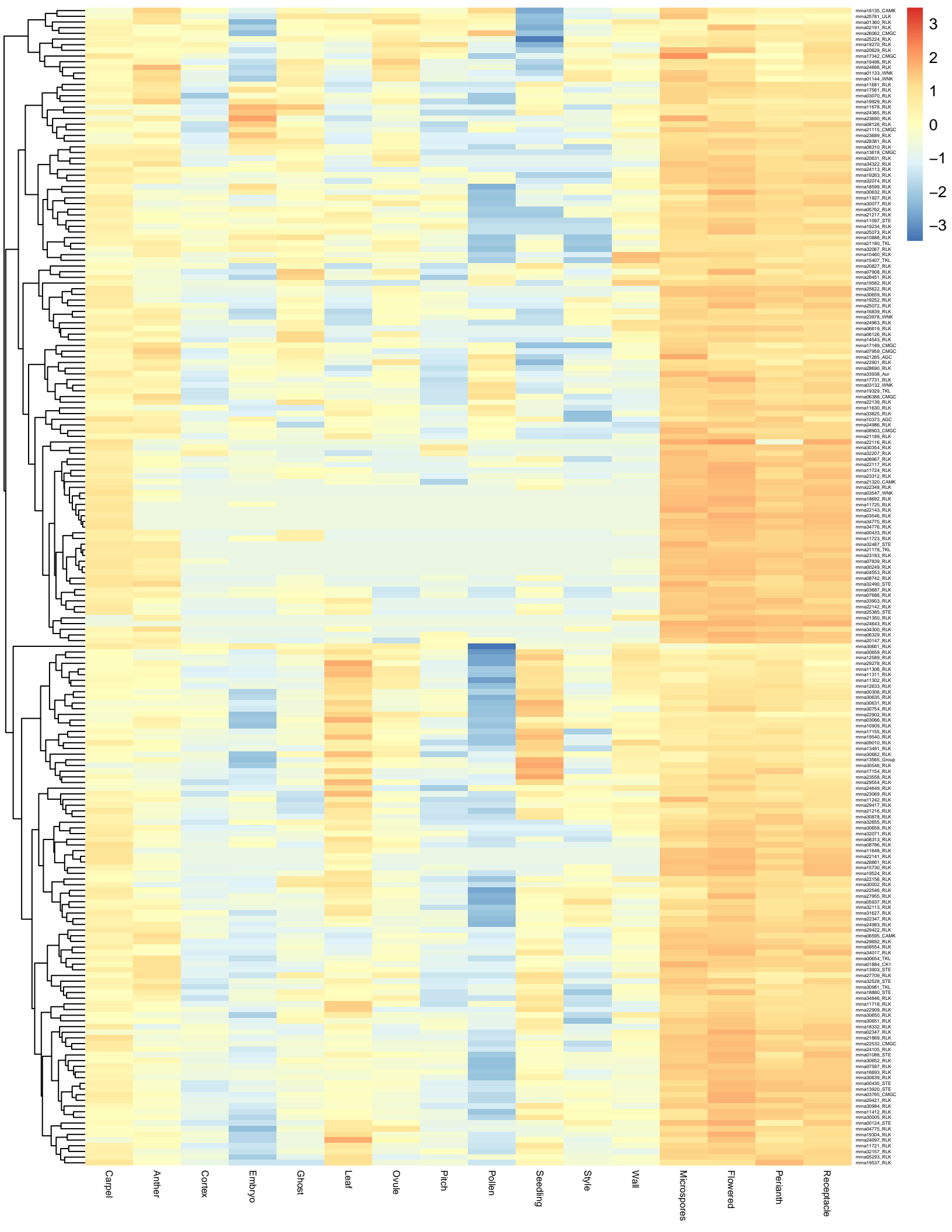

Supplement: Supplementary file 9 — Additional file 9: Figure S2. A heatmap of the expression data of strawberry kinase genes in cluster 1 in 16 different strawberry tissues and developmental stages. [file 12864_2020_7053_MOESM9_ESM.pdf]

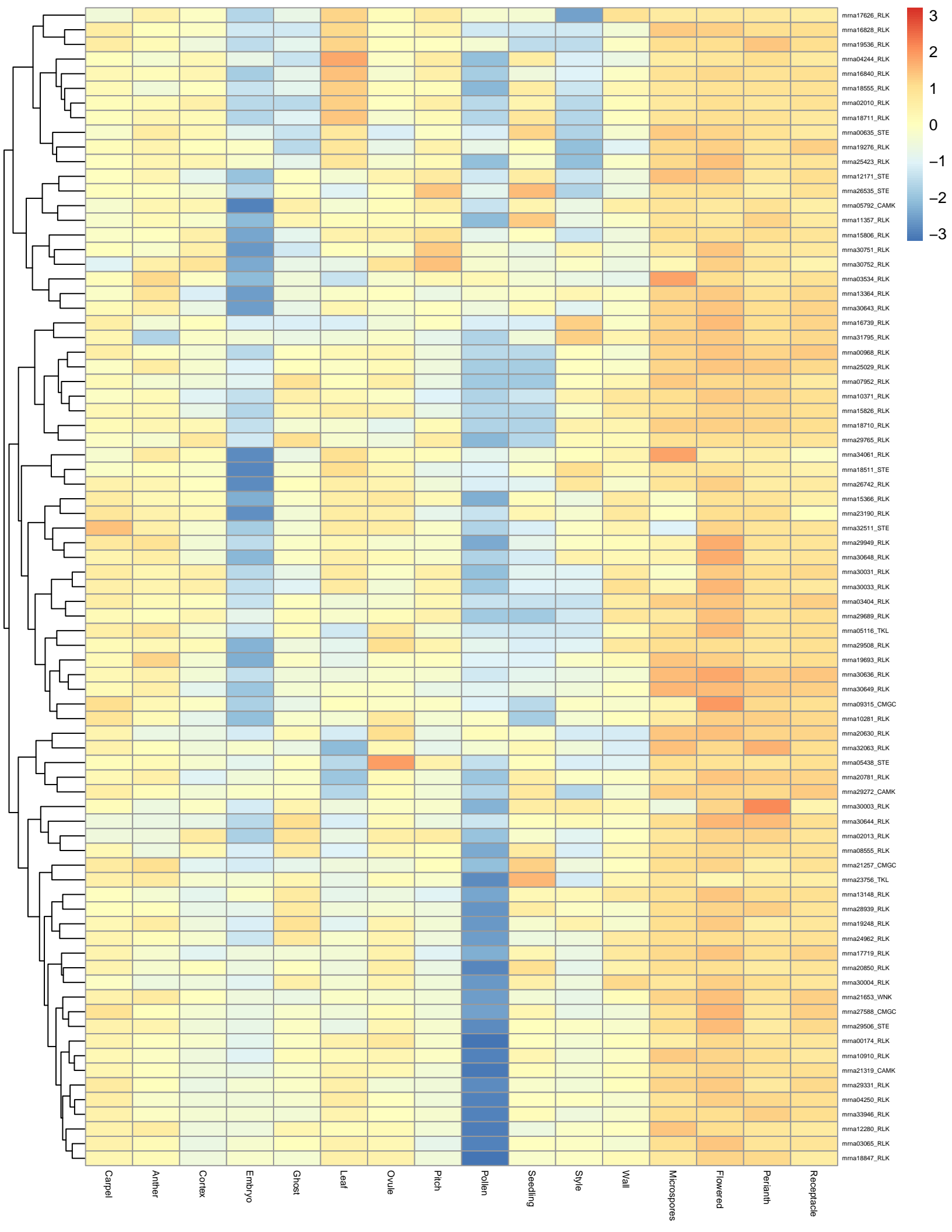

Supplement: Supplementary file 10 — Additional file 10: Figure S3. A heatmap of the expression data of strawberry kinase genes in cluster 2 in 16 different strawberry tissues and developmental stages. [file 12864_2020_7053_MOESM10_ESM.pdf]

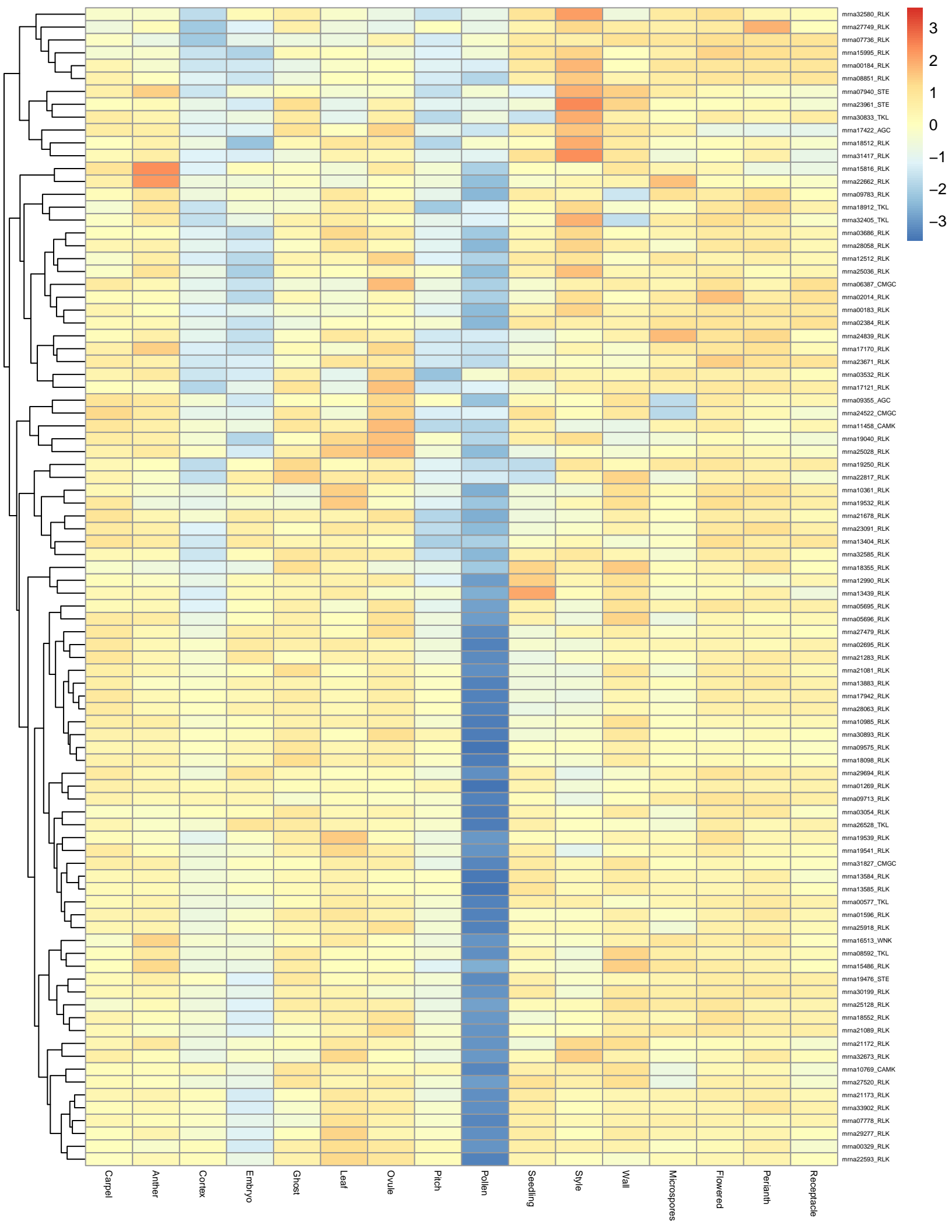

Supplement: Supplementary file 11 — Additional file 11: Figure S4. A heatmap of the expression data of strawberry kinase genes in cluster 3 in 16 different strawberry tissues and developmental stages. [file 12864_2020_7053_MOESM11_ESM.pdf]

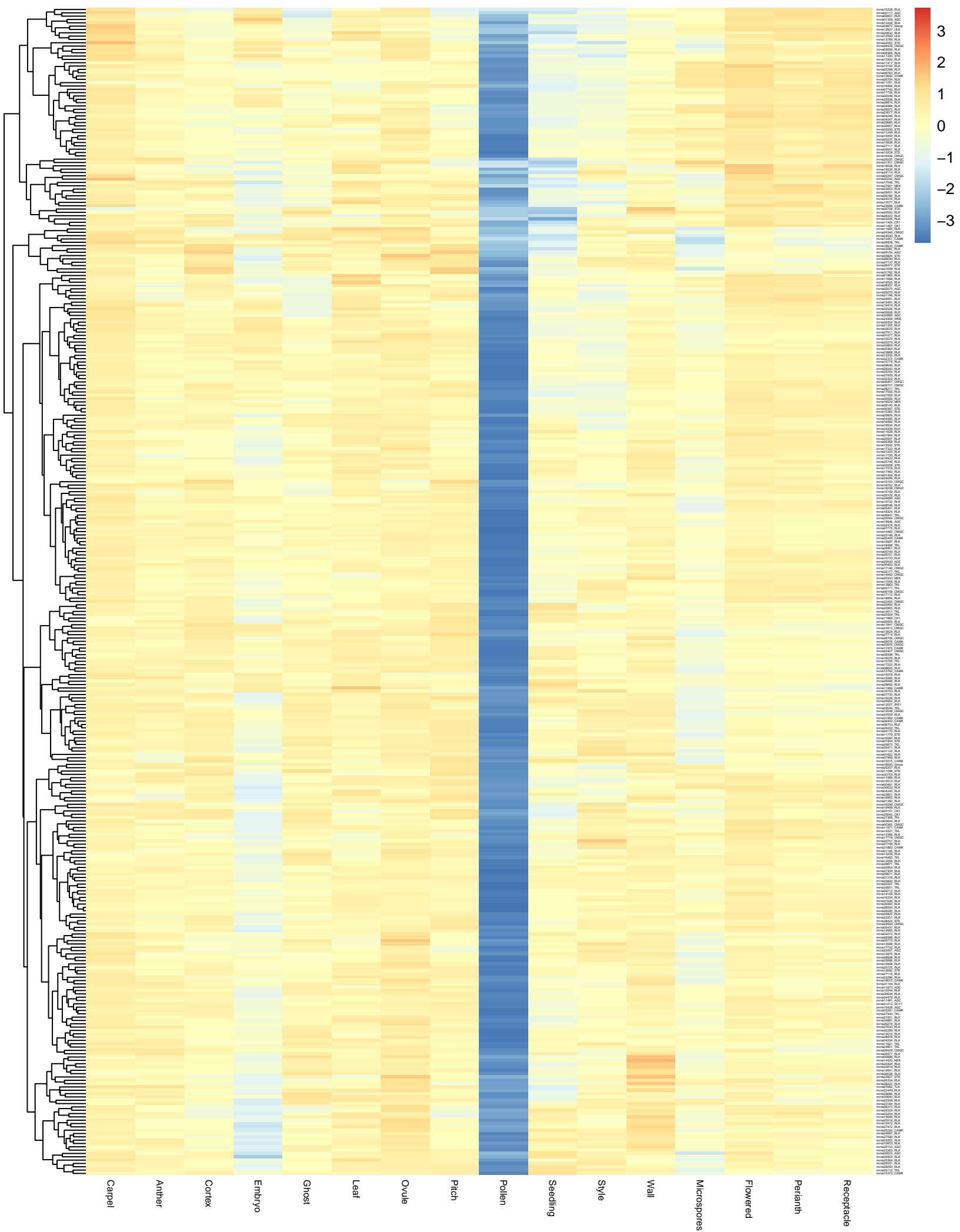

Supplement: Supplementary file 12 — Additional file 12: Figure S5. A heatmap of the expression data of strawberry kinase genes in cluster 4 in 16 different strawberry tissues and developmental stages. [file 12864_2020_7053_MOESM12_ESM.pdf]

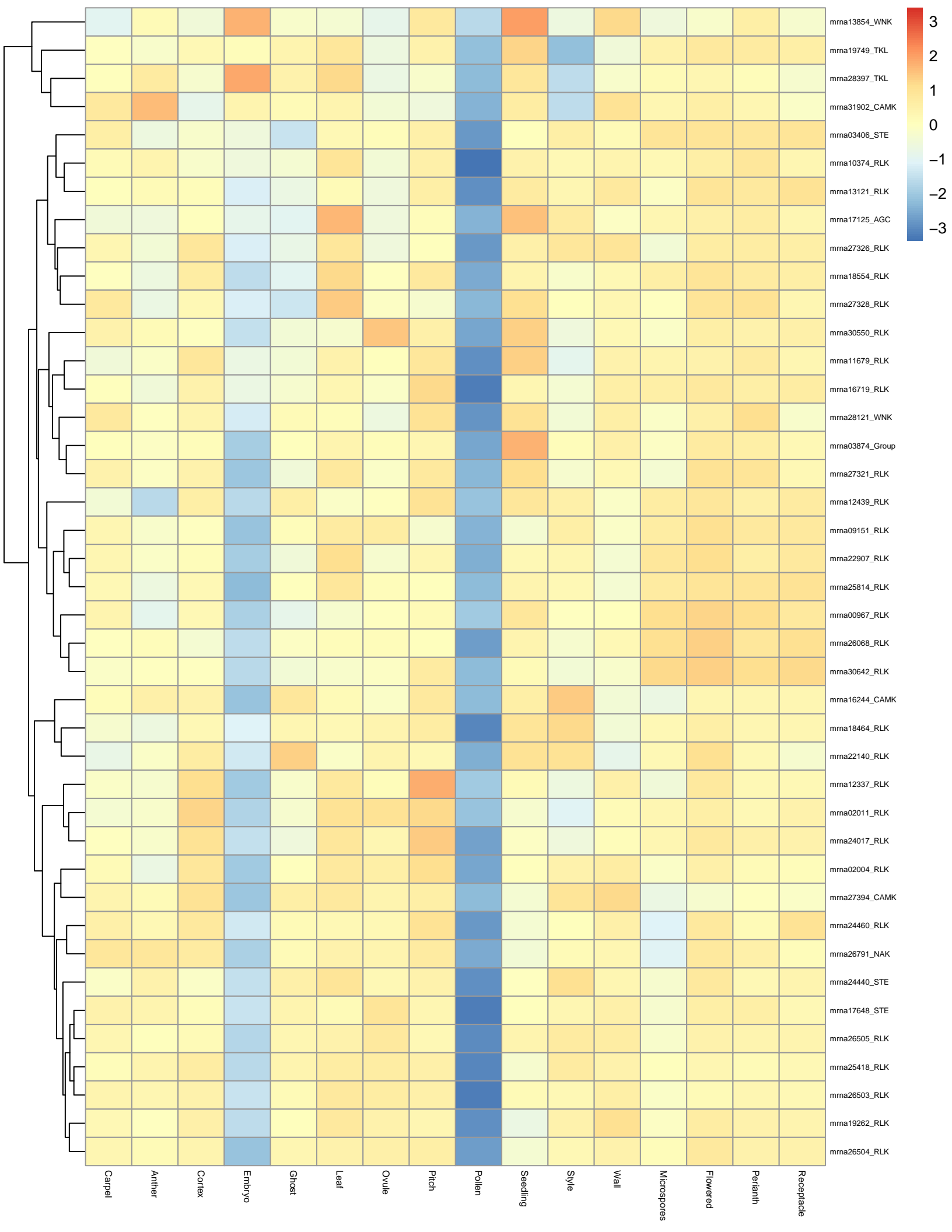

Supplement: Supplementary file 13 — Additional file 13: Figure S6. A heatmap of the expression data of strawberry kinase genes in cluster 5 in 16 different strawberry tissues and developmental stages. [file 12864_2020_7053_MOESM13_ESM.pdf]

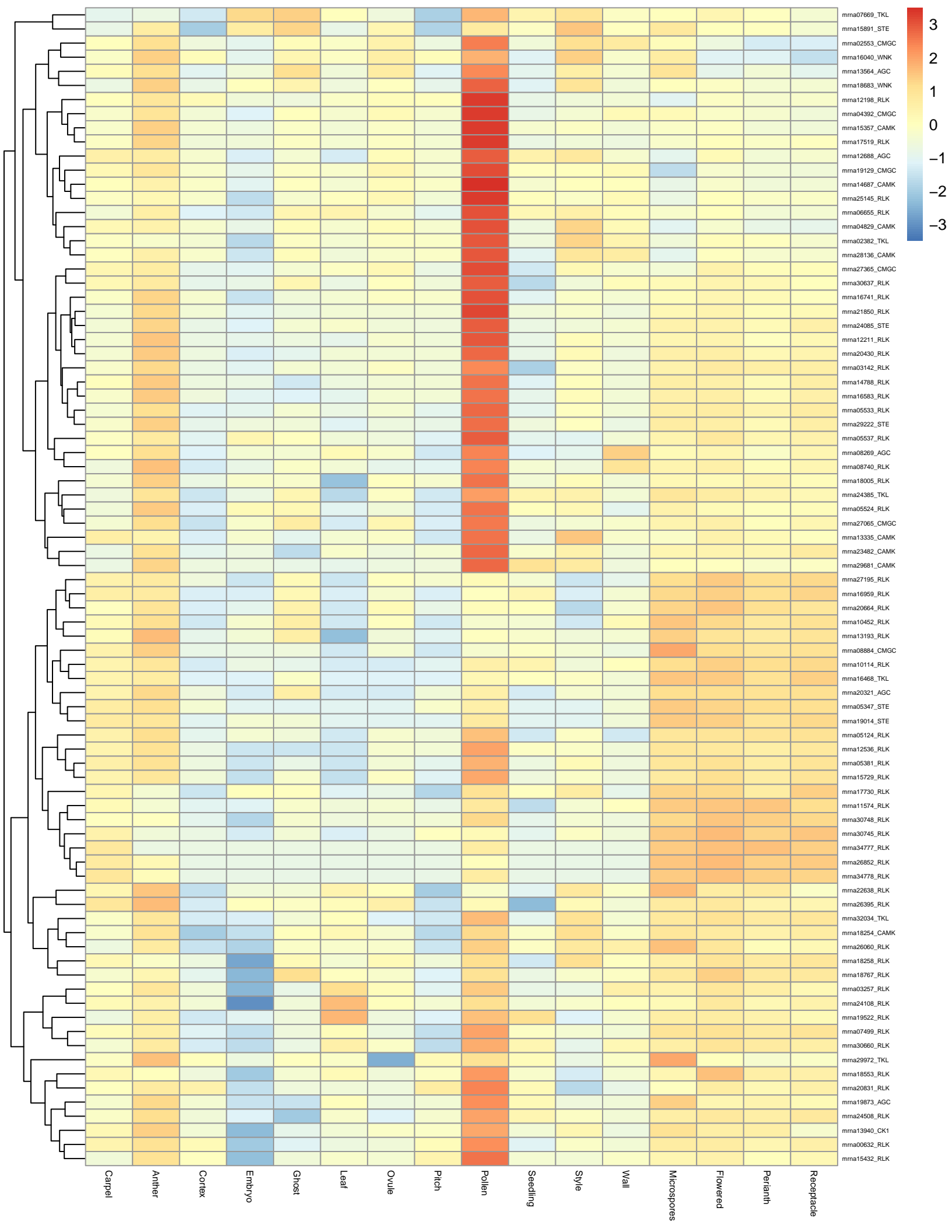

Supplement: Supplementary file 14 — Additional file 14: Figure S7. A heatmap of the expression data of strawberry kinase genes in cluster 6 in 16 different strawberry tissues and developmental stages. [file 12864_2020_7053_MOESM14_ESM.pdf]

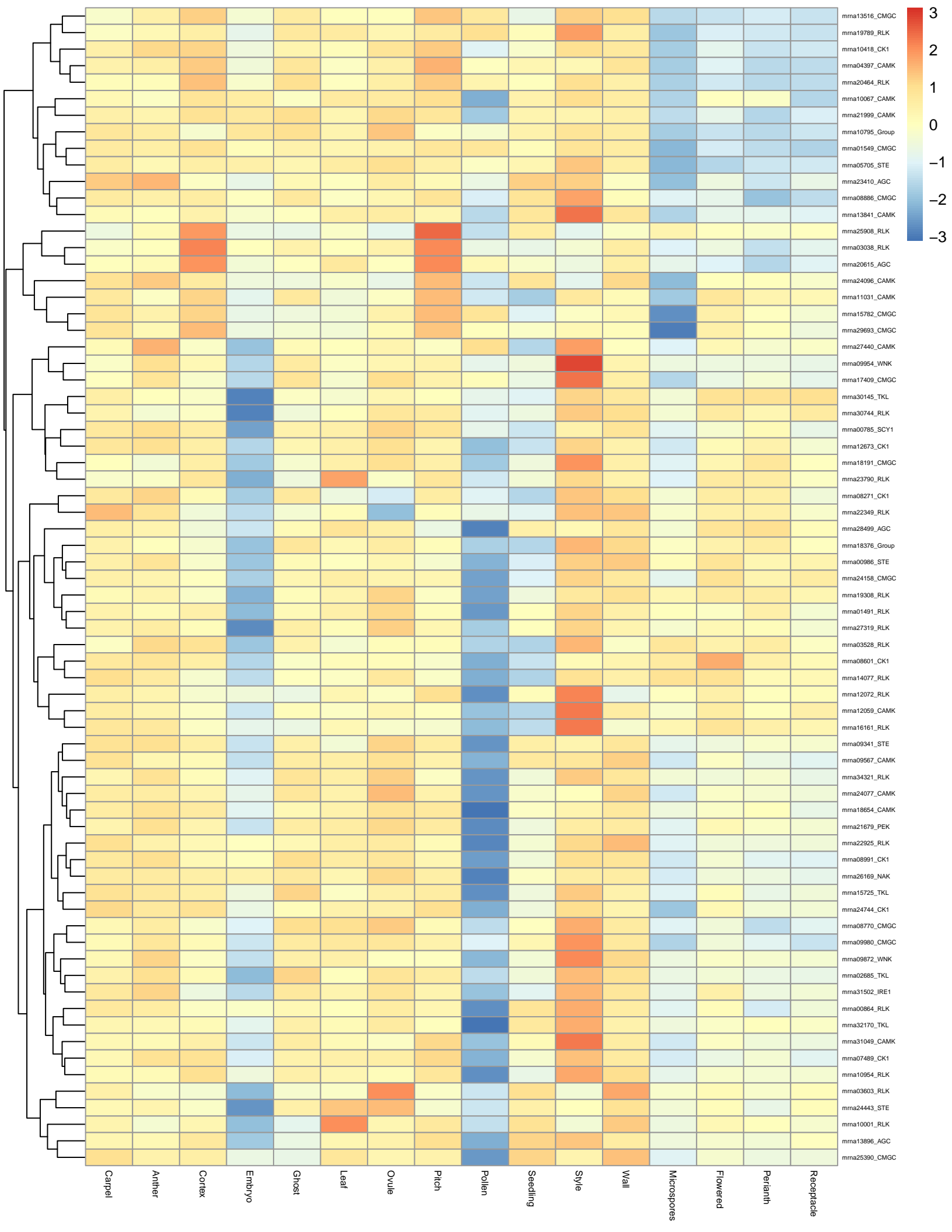

Supplement: Supplementary file 15 — Additional file 15: Figure S8. A heatmap of the expression data of strawberry kinase genes in cluster 7 in 16 different strawberry tissues and developmental stages. [file 12864_2020_7053_MOESM15_ESM.pdf]

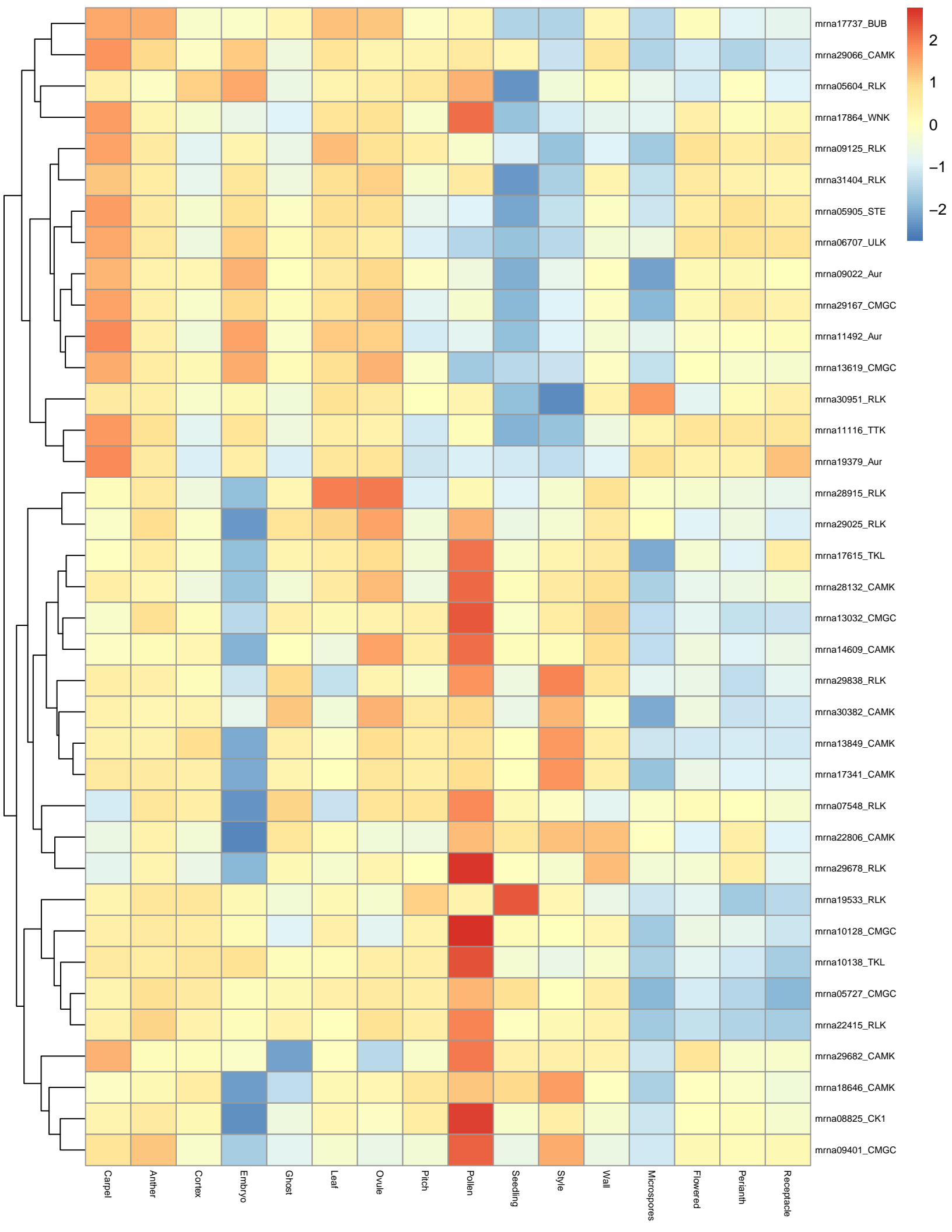

Supplement: Supplementary file 16 — Additional file 16: Figure S9. A heatmap of the expression data of strawberry kinase genes in cluster 8 in 16 different strawberry tissues and developmental stages. [file 12864_2020_7053_MOESM16_ESM.pdf]

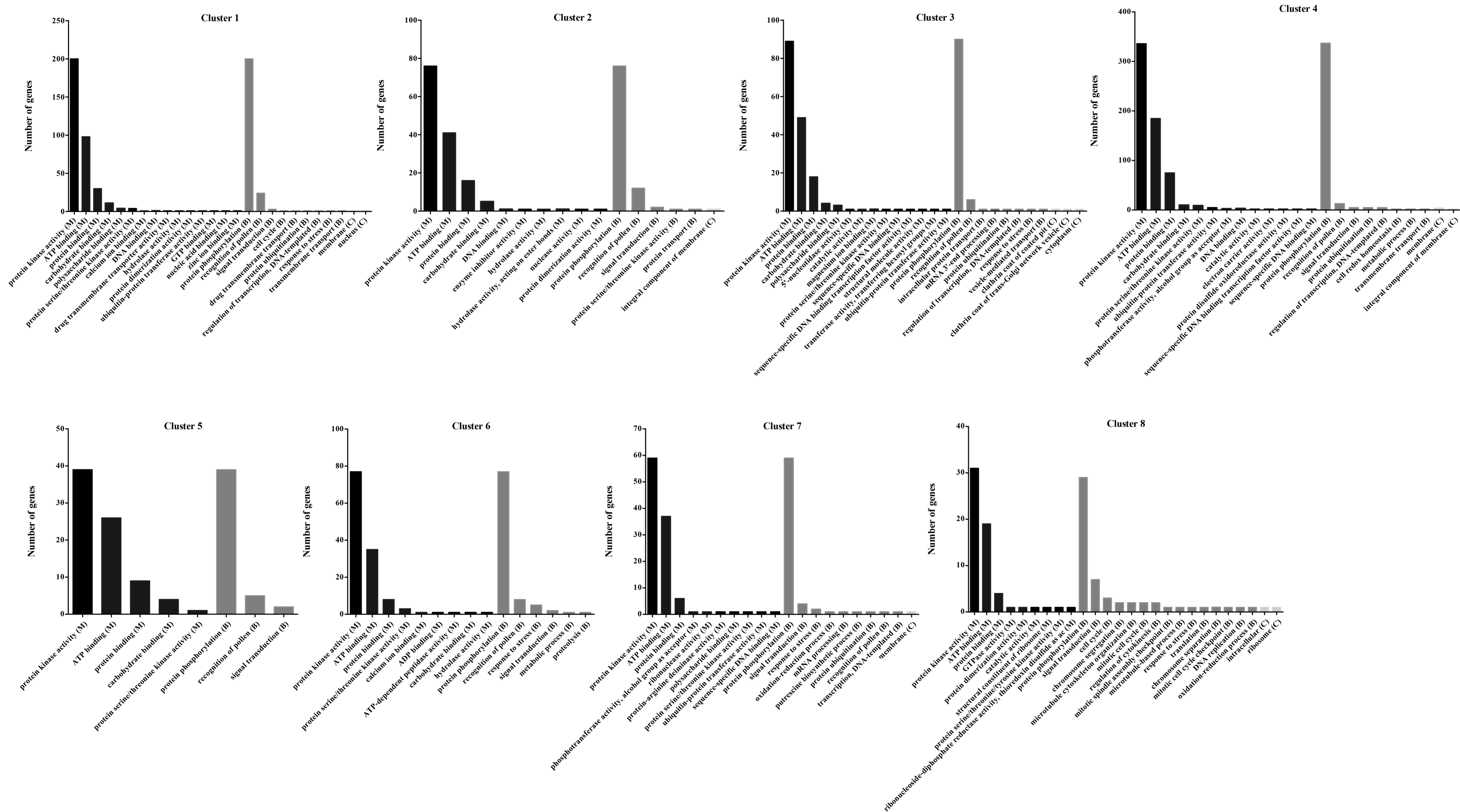

Supplement: Supplementary file 17 — Additional file 17: Figure S10. The strawberry protein kinase genes in each cluster enriched in (A) biological process (B) molecular process (C) cellular component. [file 12864_2020_7053_MOESM17_ESM.pdf]

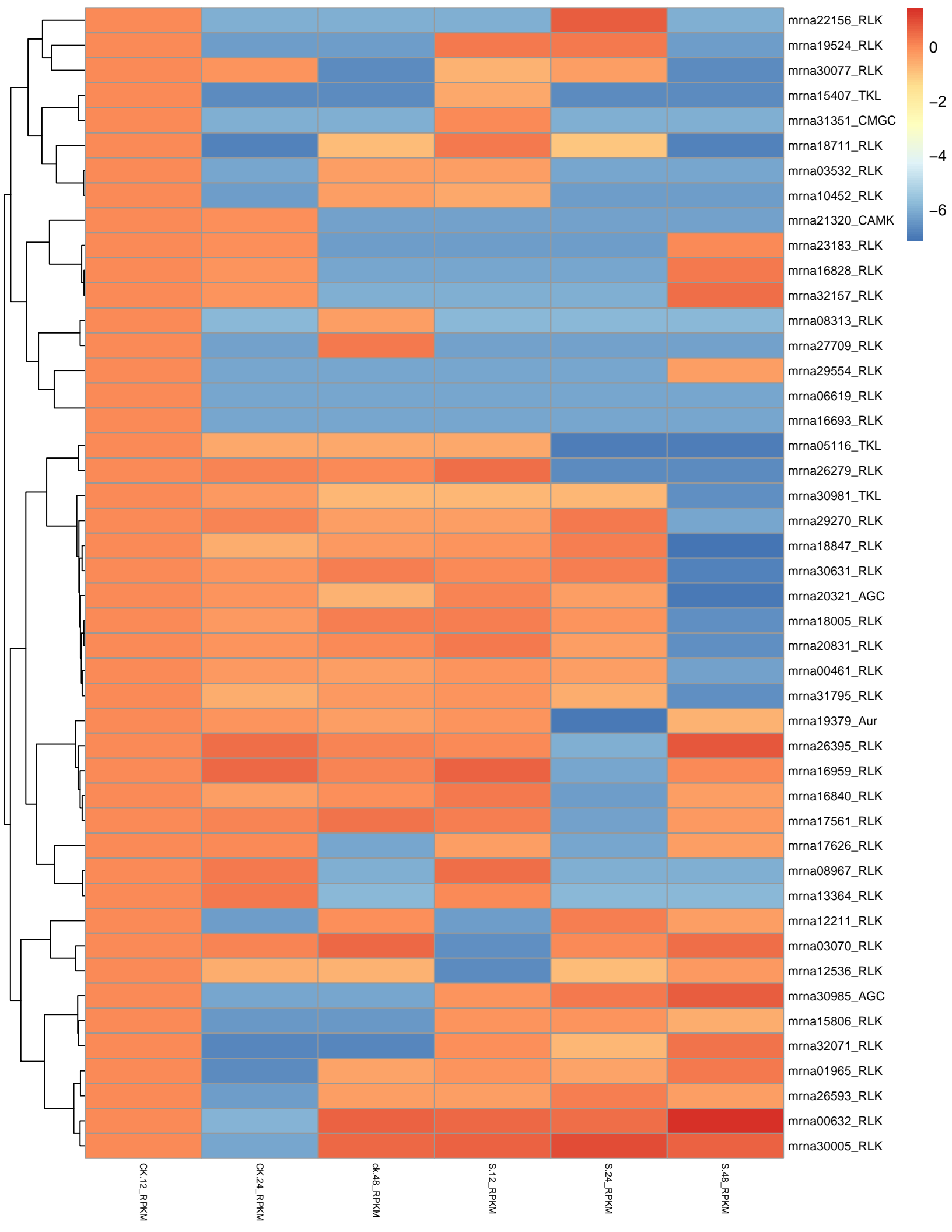

Supplement: Supplementary file 18 — Additional file 18: Figure S11. A heatmap of the expression data of the strawberry kinase genes in cluster 1 response to gray mold. [file 12864_2020_7053_MOESM18_ESM.pdf]

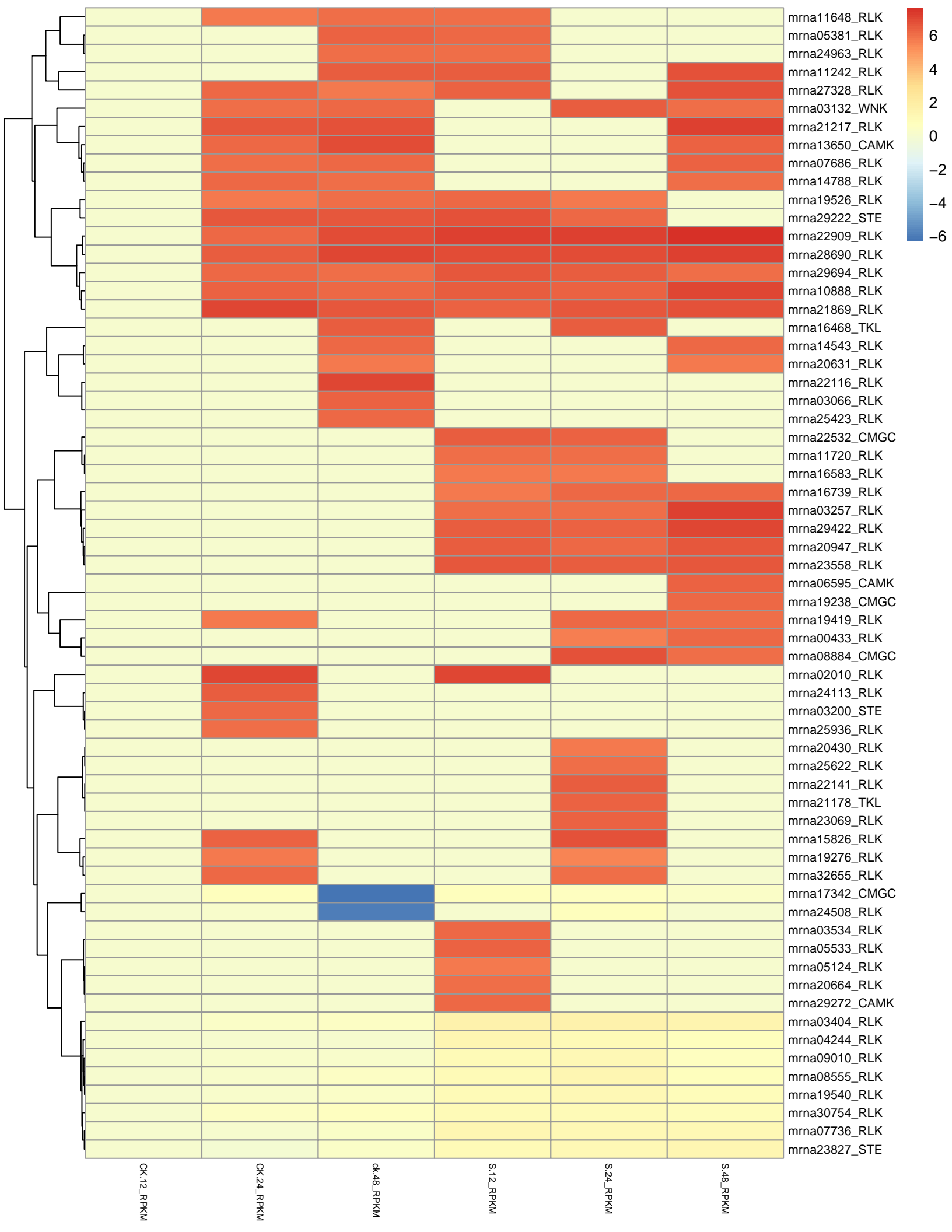

Supplement: Supplementary file 19 — Additional file 19: Figure S12. A heatmap of the expression data of the strawberry kinase genes in cluster 2 response to gray mold. [file 12864_2020_7053_MOESM19_ESM.pdf]

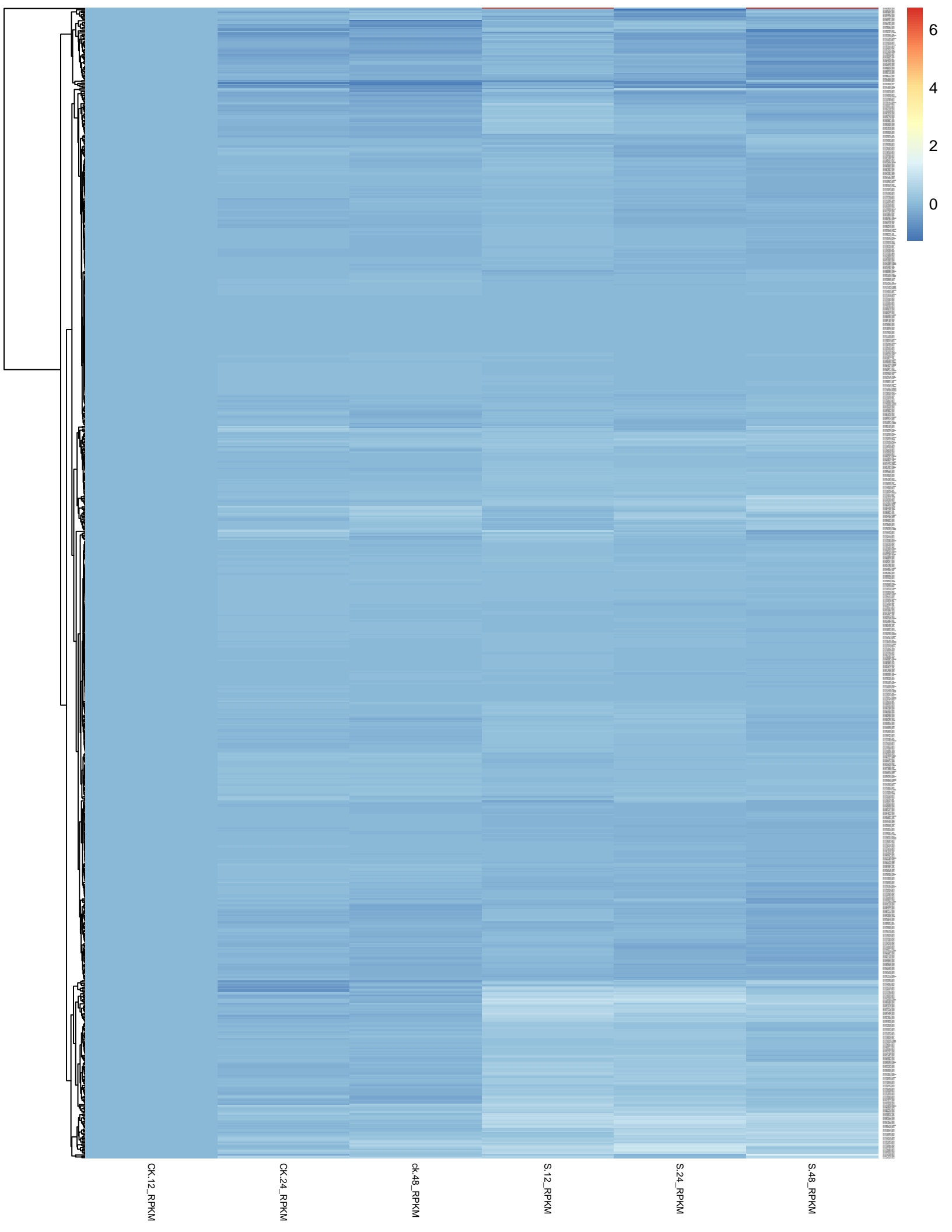

Supplement: Supplementary file 20 — Additional file 20: Figure S13. A heatmap of the expression data of the strawberry kinase genes in cluster 3 response to gray mold. [file 12864_2020_7053_MOESM20_ESM.pdf]
